# Books Reviewed

**Published:** 1867-08

**Authors:** 


### Books Reviewed.

The Science and Practice of Medicine. By William Aitkin, M. D., Edinburgh, Professor of Pathology in the Army Medical School, etc., in two volumes. From the fourth London edition, with additions, by Meredith Clymer, M. D., late Professor of the Institutes and Practice of Medicine in the University of New York, etc. Philadelphia: Lindsay & Blakiston.

In the plan of this work, the author has aimed at a fuller consideration of the pathology of disease and the philosophy of cure, than has heretofore been attempted in any of our text-books upon the Science and Practice of Medicine. In its range of topics it may be considered as *the most* comprehensive, fairly representing the existing state of medicine; while the vagaries and delusions of the past are carefully excluded from its pages. Designed especially to assist the medical student in the prosecution of his studies of diseases, the arrangement has been such, as to most fully promote this object.

The introductory sections are devoted to consideration of the more "important elements of General Pathology," and the principles upon which a nosological division of disease has been founded. The outlines of general pathology in its various relations to individual disease is investigated most comprehensively, and the intimate relationship of pathology to physiology pointed out, the author truly stating that "just in proportion as our knowledge of physiology and pathology becomes more exact and extended, so will the *cause of disease* be appreciated, and the *occurrence of disease* on a large scale prevented." Again he says: "to physiology, therefore, in its most comprehensive sense, and to a knowledge of the natural and normal development of animal and vegetable beings, we must look for a further progress in pathology, while the means and the instruments which advance physiology will simultaneously advance our knowledge regarding the *nature of diseases*—a sound knowledge of which can only enable us to *appreciate their causes*," and so arrange measures *for the prevention of many of them*, based on the great truths of science. The anatomical changes exhibited in various tissues of the body are extensively and carefully described; the author's description of amyloid or lardacious degeneration being especially attractive. This peculiar degeneration has excited a deep interest among pathologists, on account of its permanent character, undergoing no softening, exciting no inflammatory action by its presence, and being but little, if any, absorbed; any organ therefore, becoming the seat of this disorganization, is permanently impaired in its functions in proportion to the extent of the same. It would appear from Dr. Aitkins' *post-mortem* examinations, that this degeneration is of greater frequence than has generally been supposed, chiefly attacking the paranchematous structure of the liver, the spleen or the kidneys, other structures being occasionally the seat of the de generation. The train of symptoms accompanying this degeneration have not yet been fully studied, although a cachectic state of the system depending either upon necrosis, syphilis, scrofula, or phthisis, when accompanied by a great wasting of the body, the voidance of large quantities of urine, of a sp. gr. of from 1.005 to 1.015, filled with hyaline casts, should excite the suspicion of its existence, and subject the urine to a microscopical examination and the iodine test, which, in the author's opinion, can alone be positive evidence of its presence or absence. The iodine test, based by Virchow upon a supposed resemblance of the amyloid products to starch, the evidence relied upon consisting in the peculiar change which iodine undergoes when brought in contact with this product and sulphuric acid; analogous results however are obtained with cholestrine, and in view of the great uncertainty in which the whole of this subject is involved, we would incline to the belief that the iodine test is not of so much value as the author would make it appear.

The author has arranged the work into three divisions. Division first treating of systematic medicine and nosology. Class i, zymotic diseases; class ii, constitutional diseases; class iii, diseases in the course of which lesions tend to be localized; class iv, developmental diseases; class v, lesions from violence tending to sudden death.

In division second, under the head of the *Nature of Diseases*, *Special Pathology* and *Therapeutics*, the writer has described the nature of each disease, considered as characteristic of its class. In so doing the writer says, "each disease or morbid process has been defined, not by a logical definition, but merely by stating prominently its leading characters, so that the student may *at once* distinguish the general feature of the disease, which he has to study, and which the physician has to treat. Having then established the position of each disease in its *Nosological* and *Pathological* relations, those principles are stated which guide its treatment, and in some instances definite details are given."

Division third is devoted to the consideration of the *Geographical Distribution of Health and Disease.* This important study has heretofore never been introduced in any of our works on the practice of medicine, nor has hardly any allusion been made to it in the lecture rooms. The important relations which this science bears to practical medicine, cannot be overestimated, audnmuch of the value of the study of the nature of diseases and their distribution over the globe, has been lost, by not studying it, in relation to the physical condition of the earth's surface and the variation of their types in different regions of the world.

The realms or zones of diseases, the author says "are intimately associated with the temperature, and generally indicated by the regions of the tropical, temperate and polar zones;" for the better illustration of what is meant by the realms of disease, the map of Mr. Keith Johnston has been introduced. The tropical realm or zone, the author confines to Humboldt's mean annual isothermal line of 77° Fahr., north and south of the equator. The class of diseases characterizing this realm the writer considers as the most malignant forms of malarial (intermittent and remittent) fevers, associated more especially with *dysentery*, *diarrhœa*, *cholera indica*,

*specific yellow fever, hepatic affections and their results.* The paludal fevers of this tropical *disease-realm* prevail in their greatest intensity in flat, low-lying countries, in the vicinities of marshes, the borders of lakes, shores of rivers and of the sea. The maximum intensity of *dysentery, yellow fever, diarrhœa, malarial fevers, and affections of the liver*, is observed in these countries which are situated under the line of the greatest annual mean temperature, namely, 82° Fahr., which is the equator of heat of the globe," the ratio of deaths being 53 per cent. up to the 23°, while *from there to* the 35° they cause but 14 per cent. of the whole of the mortality.

The second well marked *realm* is that in which varied forms of *continued febrile* disease take the place of the *malarious* or *paludal* fever of the torrid zone. The regions where diseases of this type prevail embrace *realms* to the north and south of the equator which may be generally described as in the north and south tempertae zone. The boundary of this realm extends between the 35° and 60°, embracing the healthiest parts of the globe, the prevailing causes of ill health being mainly due to the *condensation of people in towns* and the insalubrious and depressing conditions which necessarily arise from this cause. The third disease-realm extends to the north and south of the 60° or the mean annual isothermal line of 41° Fahr. In this realm the "*catarrhal affections, influenza, scurvy, erysipelas, diseases of the skin and digestive organs, and various constitutional affections,* more especially prevail."

We have thus given our readers a general outline of the plan of Dr. Aitkin's work; a work upon which the highest encomiums of the medical press have been bestowed. The American Editor, Dr. Meredith Clymer, has made numerous additions, which greatly increase the value of a work, whose teachings may be safely followed in the treatment of disease, and which will undoubtedly influence the practice of the profession for many years.

---

On the Action of Medicines in the System. By Frederick Wm. Headland, M. D., A. B., F. L. S., Fellow of the Royal College of Physicians, etc., etc. Fifth American from the Fourth London Edition. Revised and Enlarged. Philadelphia: Lindsay & Blakiston, 1867.

"Thus have I succeeded in solving the noble problem, viz: to find a remedy for a given disease." These, the coucluding remarks of Dr. A. Pitcairn, in one of his works, serves to illustrate the almost utter ignorance, until quite lately prevalent among medical men, of the *modus operandi* of remedical agents, and no question in medicine appeared to them of an easier solution than the action and choice of a remedy in any particular case. Fortunately, these delusions have been gradually dispelled by our increased knowledge of therapeutical and physiological truths, and the questien formerly thought of an easy solution, has proved to be one of the greatest difficulty. Of late years much attention has been bestowed upon the therapeutical action of medicines, and various theories advanced, some of practical and scientific value, while others, especially those based upon the *local tendency* of medicines, are open to grave objections.

In the work under consideration, the author discusses the modes of action of therapeutical agents when introduced into the stomach, in ten propositions, in in the first four of which the general conduct of medicines after their introduction into the stomach, and before their entrance into the circulation is considered, while the remaining six, treat on the behaviour of medicines after their entrance into the fluids of the body. In the first proposition the author affirms "*that the great majority of medicines must obtain entry into the blood or internal fluids of the body, before their action can be manifested;*" which statement is proved: first, that when introduced into any other part of the body than the stomach, the action of the medicine is precisely the same; secondly, it has been ascertained by direct experiments, that a poison will not act through the medium of the nerves only, but that its passage into the blood is required; thirdly, the course of the circulation is sufficiently rapid to allow the most active poison or medicine to be absorbed and carried around the system before any of its peculiar symptoms can manifest themselves; and fourthly, with but few exceptions, all medicines have been detected, not only in the blood, but in the secretions formed from the blood. Having thus conclusively demonstrated the passage of medicines into the general circulation, the second proposition is devoted to the consideration of the laws, by which this transit is effected. The conditions necessary for the appropriation of agents so diversified in their physical properties, will be the presence of proper solvents. This condition is fulfilled for all substances, insoluble in water, either by the gastric, intestinal, biliary or pancreatic secretions, or all combined. But it has been found that a few substances are insoluble in any of the above enumerated solvents, and the question naturally suggests itself, how and in what manner do these affect the animal economy? An erroneous theory has, by some writers, been promulgated, in which they endeavor to demonstrate the actual passage of insoluble substances in a state of minute sub-division between the interstices of the animal membrane. Such a transition, the author, after careful experimental examinations, has repeatedly demonstrated to be a physical impossibility, since the highest magnifying power have failed to reveal even the minutest aperature in the prestine condition of the basement membrane; and although epithelial cells, exhibiting perforations, have been, of late, discovered by Köllicker and Virchow, the imperforate basement membrane would still bar the transition of foreign matter. In what manner, then, do insoluble medicines exert their peculiar action on the system? This query the author disposes of by stating that their action is purely local by irritation or otherwise of the mucous membrane, and that in a few instances these local actions may be succeeded by changes in distant parts, on the principle of revulsion. For example: kousso, male fern, santonine, etc., are employed as anthelmintics, which tend to directly destroy the parasite, while the carthartic given in connection is designed to expel them. Again, sulphate of zinc and copper causing, when coming in contact with the walls of the stomach, an irritation, by which the peristaltic movements of that organ is reversed, and its contents ejected without any nauseous feeling. Again, the soothing action of nitrate of bismuth, which can be administered in very large doses, (if chemically pure,) relieves the irritable condition of the alimentary tract, by affording a covering to the irritable mucous membrane, and not by any effect it exerts upon the nerves.

The author having considered the various modes in which medicines gain entrance into the fluids, devotes the remaining part of the work to their behaviour after their absorption. We should be happy to present to our readers the mode of reasoning adopted in this part of the work, but must forbear, simply stating that from a careful examination we find the reasonings singularly conclusive, and in point of originality surpassing all works upon this subject.

---

Circular No. 5, War Department, Surgeon General's Office, Washington, May 4th, 1867. Report on Epidemic Cholera in the Army of the United States during the year 1866.

Probably no question has agitated the medical mind more since the last two years than that of Epidemic Cholera, and the numerous books and pamphlets published of late, bearing upon this subject, is highly illustrative of the deep interest felt on the part of the medical profession. With praise-worthy energy Brevet Lieut. Col. J. J. Woodward, Assistant Surgeon U. S. A., devoted himself to the arduous and difficult task of preparing a "Report on Epidemic Cholera," as it occurred in the United States Army during the year 1866, and the history therein transmitted by him to the Surgeon General is entitled to the consideration of all interested in this subject.

From the monthly reports transmitted from each station it appears that out of a total mean strength of 12,780 men 2,708 attacks of cholera had occurred with a fatality of 1,207, or nearly 50 per cent. of deaths of all attacked by the disease. Out of the total number of attacks, 1,749 cases with 706 deaths, occurred among the white troops, with a mean strength of 9,083 men; the remaining 959 attacks with 501 deaths occurring in a mean strength of 3,697 colored troops. The maximum fatality seems to have been on the first and second days after the attack recoveries becoming proportionately more frequent as the disease progressed, the greatest duration of any fatal case being to the fifteenth day.

Regarding the origin and spread of the disease, Dr. Woodward says: "The epidemic appears from the records to have radiated from two chief centers." The first "originating in the overcrowded barracks of Governor's Island, New York harbor, in the immediate vicinity of an infected city, through which recruits passed with more or less delay before arrival. The infection spread by traceable steps to Hart's Island and other points in the harbor, to Tybee Island, Georgia; to Louisiana, by way of New Orleans; to Texas by way of Galveston, etc. The other principal center appears to have been Newport barracks, Kentucky, where the disease was plainly introduced from the infected city of Cincinnati, on the opposite side of the Ohio river, spreading from thence to Augusta and Atlanta, Ga., and Nashville and Memphis, Tenn."

The value of stringent hygienic precautionary measures is strikingly illustrated in the reports of Brevet Major E. McClellan, Asst. Surgeon U. S. Army, in charge of Fort Delaware, who says: "Epidemic cholera has existed to a very considerable extent during the past month in, and around Delaware City, where, in the aggregate of fifteen hundred inhabitants, some thirty odd deaths have occurred. No case has occurred among the troops of this command, and but one upon the island. The most rigid regulations are enforced as regards communication with either of the adjacent towns, and no fruit is permitted to be landed upon the island." From this and other similar statements of surgeons, it would appear that quarantine exerted a great influence in arresting the disease, and although the question is not conclusively proved, the facts nevertheless are of great significance. Hygienic measures greatly mitigate the severity of the disease making it more amenable to treatment. No new therapeutic methods have been suggested which possess any value, the results being alike, all things equal, no matter what therapeutical agents are employed.

